# Breaking data silos: incorporating the DICOM imaging standard into the OMOP CDM to enable multimodal research

**DOI:** 10.1093/jamia/ocaf091

**Published:** 2025-07-18

**Authors:** Woo Yeon Park, Teri Sippel Schmidt, Gabriel Salvador, Kevin O’Donnell, Brad Genereaux, Kyulee Jeon, Seng Chan You, Blake E Dewey, Paul Nagy, Alzheimer’s Disease Neuroimaging Initiative, Woo Yeon Park, Teri Sippel Schmidt, Gabriel Salvador, Kevin O’Donnell, Brad Genereaux, Kyulee Jeon, Seng Chan You, Blake E Dewey, Paul Nagy

**Affiliations:** Biomedical Informatics and Data Science, Johns Hopkins University, Baltimore, MD 21205, United States; Biomedical Informatics and Data Science, Johns Hopkins University, Baltimore, MD 21205, United States; Biomedical Informatics and Data Science, Johns Hopkins University, Baltimore, MD 21205, United States; Canon Medical Research United States Inc., Vernon Hills, IL 60061, United States; Biomedical Informatics and Data Science, Johns Hopkins University, Baltimore, MD 21205, United States; NVIDIA Corporation, Santa Clara, CA 95051, United States; Department of Biomedical Systems Informatics, Yonsei University College of Medicine, Seoul 03722, Republic of Korea; Institute for Innovation in Digital Healthcare, Yonsei University Health System, Seoul 03722, Republic of Korea; Department of Biomedical Systems Informatics, Yonsei University College of Medicine, Seoul 03722, Republic of Korea; Institute for Innovation in Digital Healthcare, Yonsei University Health System, Seoul 03722, Republic of Korea; Biomedical Informatics and Data Science, Johns Hopkins University, Baltimore, MD 21205, United States; Department of Neurology, Johns Hopkins University, Baltimore, MD 21287, United States; Biomedical Informatics and Data Science, Johns Hopkins University, Baltimore, MD 21205, United States

**Keywords:** DICOM, OMOP CDM, multimodal data, standardization

## Abstract

**Objective:**

This work incorporates the Digital Imaging Communications in Medicine (DICOM) Standard into the Observational Medical Outcomes Partnership Common Data Model (OMOP CDM) to standardize and accurately represent imaging studies, such as acquisition parameters, in multimodal research studies.

**Materials and Methods:**

DICOM is the internationally adopted standard that defines entities and relationships for biomedical imaging data used for clinical imaging studies. Most of the complexity in the DICOM data structure centers around the metadata. This metadata contains information about the patient and the modality acquisition parameters. We parsed the DICOM vocabularies in Parts 3, 6, and 16 to obtain structured metadata definitions and added these as custom concepts in the OMOP CDM vocabulary. To validate our pipeline, we harvested and transformed DICOM metadata from magnetic resonance images in the Alzheimer’s Disease Neuroimaging Initiative (ADNI) study.

**Results:**

We extracted and added 5183 attributes and 3628 coded values from the DICOM standard as custom concepts to the OMOP CDM vocabulary. We ingested 545 ADNI imaging studies containing 4756 series and harvested 691 224 metadata values. They were filtered, transformed, and loaded in the OMOP CDM imaging extension using the OMOP concepts for the DICOM attributes and values.

**Discussion:**

This work is adaptable to clinical DICOM data. Future work will validate scalability and incorporate outcomes from automated analysis to provide a complete characterization research study within the OMOP framework.

**Conclusion:**

The incorporation of medical imaging into clinical observational studies has been a barrier to multi model research. This work demonstrates detailed phenotypes and paves the way for observational multimodal research.

## Introduction

### Background and significance

Observational research relies on data collected from clinical data sources to generate evidence driven medical advances. A primary source of this data is the Electronic Health Record (EHR). However, the structured data extracted from EHRs do not create a comprehensive patient picture, with valuable insights left in unstructured data.^1^^,^^2^ Healthcare utilizes many modalities of data, including structured tables, images, waveforms, and narrative texts. This multimodality poses various challenges for researchers, such as data processing and knowledge abstraction.^3–6^ In this work, we focus on surmounting the problem of integrating medical imaging data with structured EHR data.

Medical images are large binary files in a Picture Archive and Communication System (PACS) or Vendor Neutral Archive (VNA) using the Digital Imaging Communications in Medicine (DICOM) format. The DICOM Standard specifies interoperable protocols, image format, and file structure for biomedical images and imaging-related information.^7^ It includes a dictionary that defines imaging studies and standardizes the metadata in “Attributes” alongside their permitted values known as “Value Sets.” While DICOM is a globally adopted standard in clinical imaging workflows, its structured metadata, such as imaging acquisition parameters, is stored outside the central EHR system. When conducting observational research using EHR-based data, researchers lack access to detailed imaging features due to this data silo. Our approach is focused on bridging the gap in characterizing medical images by integrating the DICOM Standard and standardizing imaging information alongside structured EHR data for multimodal observational research.

The Observational Medical Outcomes Partnership Common Data Model (OMOP CDM) is a data standard developed by the Observational Health Data Science and Informatics (OHDSI) community to harmonize health data for observational outcomes research.^8^ The international OHDSI community consists of over 4200 collaborators with 810 million unique patients records organized in the OMOP CDM.^9^ OMOP CDM is composed of 39 tables, categorized in six standardized domains: clinical data, health system, vocabularies, health economics, derived elements, and metadata. It is mainly used to standardize EHR or claims databases. It is a federated framework where each institution extracts, loads and transforms their data into OMOP CDM tables and vocabulary, and only the phenotype definitions or analytical methods are shared, preserving patient’s privacy and raw data.^10^ Leveraging CDMs is a way to enhance reproducibility among multiple data sources and across institutions. A study evaluated multiple CDMs on six criteria—content coverage, integrity, flexibility, ease of querying, standards compatibility, and ease and extent of implement, and concluded that OMOP CDM met all the criteria.^11^ Additionally, its open-source software and analytical infrastructures made it a compelling base model for building multimodal standardization.^12^

Extensions are used by domain specific workgroup at OHDSI to represent specific data elements that are not currently part of the canonical OMOP CDM.^13^ The authors of this study are members of the OHDSI Medical Imaging Workgroup. The OMOP CDM currently represents images as only imaging procedures. This study strives to standardize detailed information on the medical images, such as acquisition parameters or imaging findings, to integrate information in imaging studies with EHR variables. The workgroup first developed the radiology extension (R-CDM) and most recently released medical imaging extension (MI-CDM) to encompass all types of medical images beyond radiology.^14^ The MI-CDM added two new tables—Image_occurrence and Image_feature—to incorporate details about medical images. In this paper, we focused on the semantic integration necessary to incorporate DICOM as a vocabulary that can be used in OMOP research.

### Related works

Integrating imaging data into standardized, structured EHR data requires structural and semantic standardization.^15^ The structural aspect was addressed by the two tables added through MI-CDM. The semantic standardization better describes clinical observations, diagnosis, and procedures. Other related works have provided utilities, ontologies, and terminologies to support imaging research; however, approaches that address structure and semantics separately fall short of achieving full data standardization needed for reproducible multimodal research.

Structural solutions have been developed for specific modalities or research support systems. The Brain Imaging Data Structure (BIDS) is a standard for organizing and describing neuroimaging datasets.^16^ This structure is optimized for organizing image files to advance data processing operations for neuroscience research. It primarily utilizes NIfTI (Neuroimaging Informatics Technology Initiative) files and includes a limited set of metadata.^17^ In contrast, we aimed to develop generalizable solution for organizing and representing events and features for all imaging modality and to integrate them with structured EHR data. Other systems worked to connect the PACS and clinical databases through data architectures.^18^^,^^19^ Kaspar et al developed a PACS-to-Data Warehouse (P2D) system to extract all metadata from images stored in PACS and integrate them with structured data in the clinical data warehouse.^18^ Their approach involved creating a new user interface that indexed both the DICOM images and clinical data warehouse. In contrast, our work focused on leveraging the existing OMOP infrastructure to create computable phenotypes using imaging characteristics, without requiring additional interfaces or indexing systems. Almeida et al proposed an indexing and query interface within the Dicoogle Plugin, a PACS plugin architecture, allowing researchers to define cohorts from OMOP CDM and can be used to extract the relevant medical images based on a cohort definition.^19^ While this approach aligns more closely with our goals, a key distinction lies in how we represent imaging metadata. Instead of embedding the metadata in the Observation table, we integrated DICOM terminology as OMOP concepts—the code mapping in the OMOP vocabulary—within the imaging extension model. This approach ensures that imaging metadata is semantically aligned with DICOM Standard and is consistently represented in the OMOP framework.

There are also ontologies and terminologies that describe imaging studies and findings from the semantic aspect. The Radiology Lexicon (RadLex) was developed by the Radiological Society of North America (RSNA) and American College of Radiology (ACR) to describe relevant anatomy, diseases, imaging findings, procedures, and other common concepts used in radiology.^20^ The Radiology common data elements (CDEs), or RadElement, are questions and allowable answers to express observations in radiology-specific diagnoses.^21^^,^^22^ It was developed using RadLex and other controlled medical terminologies. The Radiology Gamuts Ontology (RGO) is a knowledge model of differential diagnosis in radiology.^23^ Magnetic Resonance Imaging Acquisition and Analysis Ontology (MRIO) is developed to characterize MRI acquisition and analysis.^24^ They created new terminology to describe imaging as well as relationships between terms. At the feature level, the Image Biomarker Standardization Initiative (IBSI) proposed a list of reproducible radiomic features to standardize imaging biomarkers for analysis.^25^ Domain-specific ontologies include Mindboggling morphometry.^26^ The Mindboggling morphometry platform converts preprocessed T1-weighted MRI data into volume, surface, and tabular data. These semantic variations are useful, but they are developed for specific modality or domain (eg, MRIO, Mindboggling) and interpretation of imaging findings (eg, RadLex, RadElement, RGO, IBSI). This work focused on how the imaging studies were acquired and accurately characterize them, regardless of modality or domain, in a standardized data model.

### Interoperability standards and MI-CDM

Bridging the gap in clinical practice between EHR data and imaging data stored in PACS has been partially addressed by established healthcare interoperability standards, including the U.S. Core Data for Interoperability (USCDI), the U.S. Core Implementation Guide, and Fast Healthcare Interoperability Resources (FHIR). USCDI v5 includes the Diagnostic Imaging Test and Diagnostic Imaging Report as the data element.^27^ Additionally, two imaging-related elements, Accession Number (the imaging procedure order number recorded in the EHR and referenced in DICOM PS3.4 Section C.6.2.1, tag 0008,0050) and the Imaging Reference Number (corresponding to DICOM Unique Identifiers used to retrieve specific studies, series, or instances)—are currently classified as Level 2 and have not yet been included in USCDI.^28^ Level 2 indicates that the elements are mature, well-supported and will be considered toward the USCDI inclusion.

Similarly, FHIR R4 defines the ImagingStudy resource, adopting the hierarchical structure of the DICOM model (study-series-instance) and mapping directly to DICOM-defined attributes.^29^ The US Core Implementation Guide aligns imaging procedures under the Procedure resource profile, leveraging standard terminologies such as LOINC.^30^ The existing OMOP CDM Procedure_occurrence table is consistent with this approach, capturing procedure-level data. FHIR R5 adds the Imaging Selection Resource to identify specific images or regions in clinical practice.

The MI-CDM was designed to be fully compatible with these interoperability frameworks but serves a distinct purpose. While standards like USCDI and FHIR are intended to support clinical interoperability and facilitate real-time data exchange within and across healthcare systems, MI-CDM focuses on research-oriented use cases that require a more comprehensive capture of DICOM metadata. These include imaging and results provenance, modality-specific acquisition parameters, and study-level contextual metadata that are often critical for imaging-based phenotyping, retrospective cohort studies, and multimodal analyses. In this way, MI-CDM leverages existing interoperability standards by incorporating their utility for the secondary use of imaging data in research contexts.

### Objective

This work aims to incorporate DICOM terminology into OMOP CDM vocabulary, enabling the accurate representation of medical images for observational research. This is demonstrated through a use case from the Alzheimer’s Diseases Neuroimaging Initiatives (ADNI).

## Materials and methods

### DICOM terminology and MI-CDM

DICOM 3.0 is an internationally adopted standard that defines the entities and relationships of biomedical imaging data, such as equipment, acquisitions, and images. It is widely used in radiology and is increasingly adopted by other specialties like cardiology, pathology, and ophthalmology.^31–34^ The DICOM metadata captures detailed image acquisition parameters that are not typically available in the EHR but are critical for distinguishing imaging protocols. For example, while the EHR may contain an order for a “Brain MRI,” this level of information is typically sufficient for scheduling and billing but lacks the detail required to identify the imaging protocol. In clinical practice, the radiologist specifies detailed acquisition parameters, such as requesting a T1-weighted scan, which is characterized by a short Repetition Time (TR) and Echo Time (TE), compared to T2-weighted or FLAIR series.^35^ These parameters, along with others such as field strength and manufacturer model, are captured in the DICOM metadata and are crucial not only for clinical interpretation but also to identify variability and reproducibility in imaging research and the application of image-processing algorithms optimized for specific protocols.

DICOM data is organized in a hierarchical structure consisting of patient, study, series, and instance levels. Each level includes a set of standard metadata, referred to as “Attributes,” in a key-value format.^36^ The keys, known as “Tags,” are defined in Part 6 (Data Dictionary) of the DICOM Standard. For some attributes, Part 16 (Content Mapping Resource) specifies allowable coded values, referred to as “Value Sets” or “Context Groups”. These have specified semantics and are encoded as a triplet: Code Value, Coding System, and Code Meaning. The codes in a Context Group are often drawn from standardized terminologies, such as Systematized Medical Nomenclature for Medicine-Clinical Terminology (SNOMED) and Logical Observation Identifiers Names (LOINC), but also include codes defined and managed by DICOM.^37^ For other attributes, allowable coded values are defined in Part 3, called “Enumerated Values” or “Defined Terms,” which specify the semantics in the context of that attribute. Example tables from the DICOM Standard are provided in Appendix S1.

**Figure 1.**
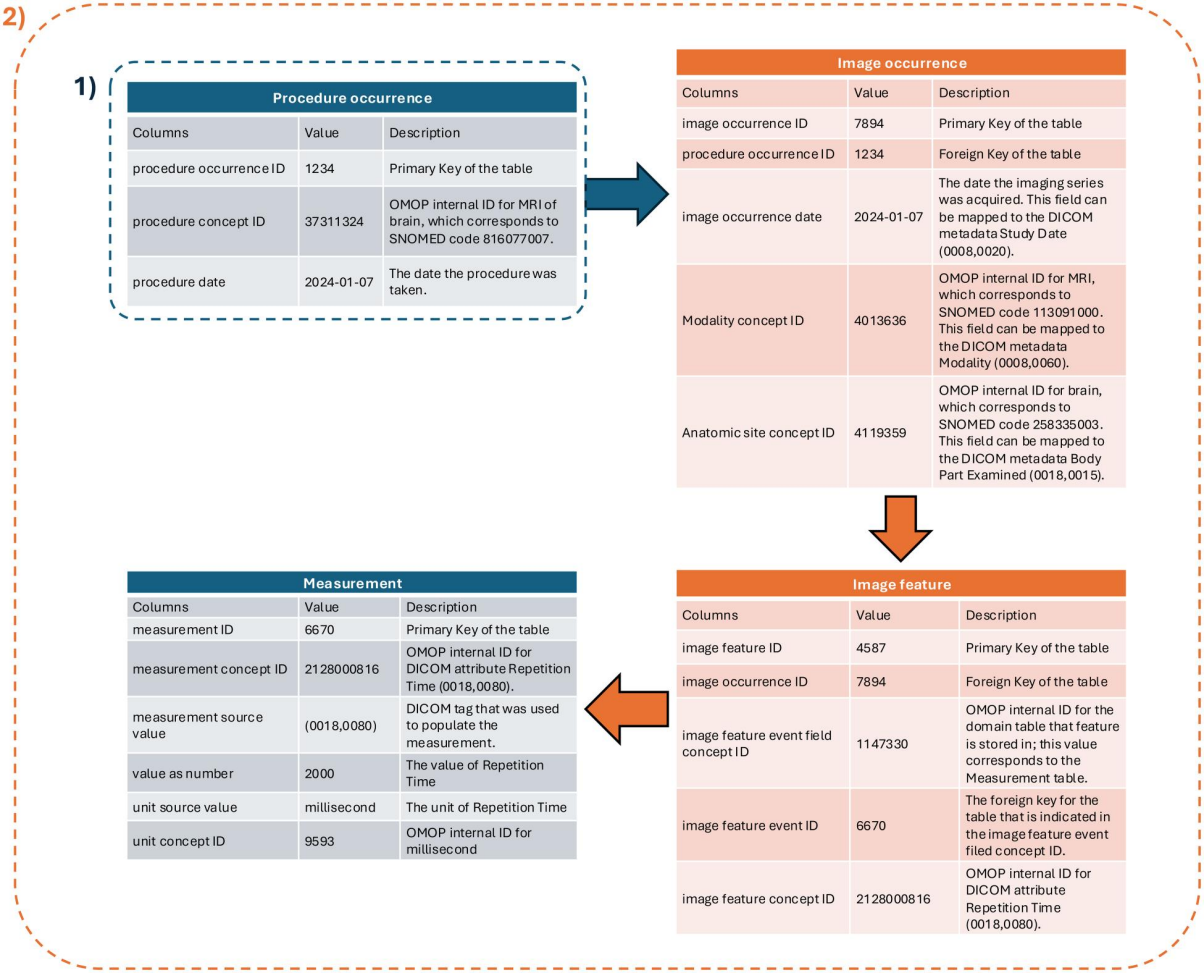
An example of OMOP CDM and MI-CDM on imaging study data representation.

To address this limitation, we extracted DICOM terminology, integrating it into the OMOP CDM vocabulary and enabling the MI-CDM to capture and characterize this essential imaging metadata within existing OMOP CDM conventions. A comparison between standard procedure-level representation and the MI-CDM metadata elements is summarized in Figure 1.

### Method overview

**Figure 2.**
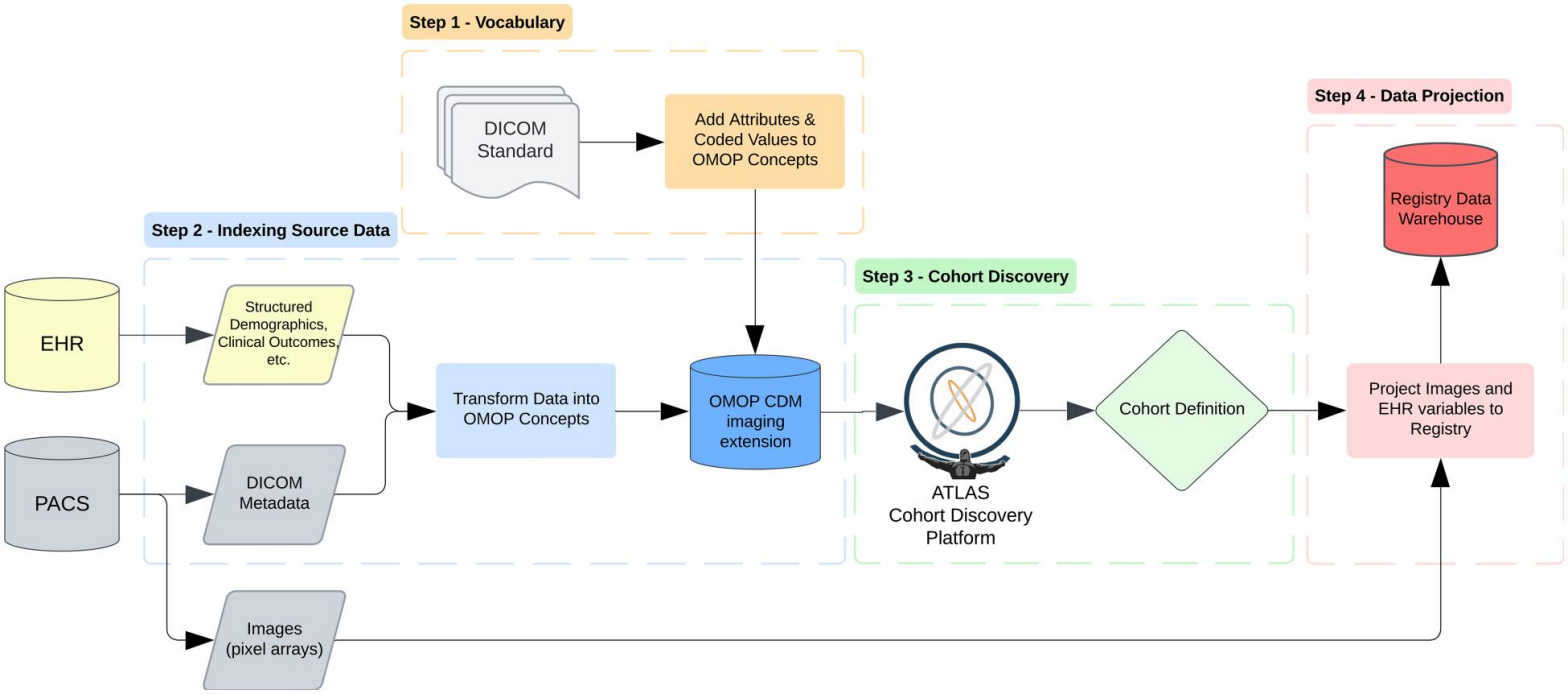
Overview of the study method in four steps.

This work was executed in four steps from vocabulary development to practical application (Figure 2). The vocabulary development involved extracting and preprocessing DICOM Standard terms for incorporation into the OMOP vocabulary. After the DICOM terms were incorporated, the practical application included indexing source data, cohort discovery, and data projection. As a demonstration, we ingested data from the ADNI research study and used ATLAS, an analytic platform developed by OHDSI, to query and formulate cohort definitions that can help to answer real-world research questions.

### Extracting the DICOM standardized elements and adding DICOM concepts

**Figure 3.**
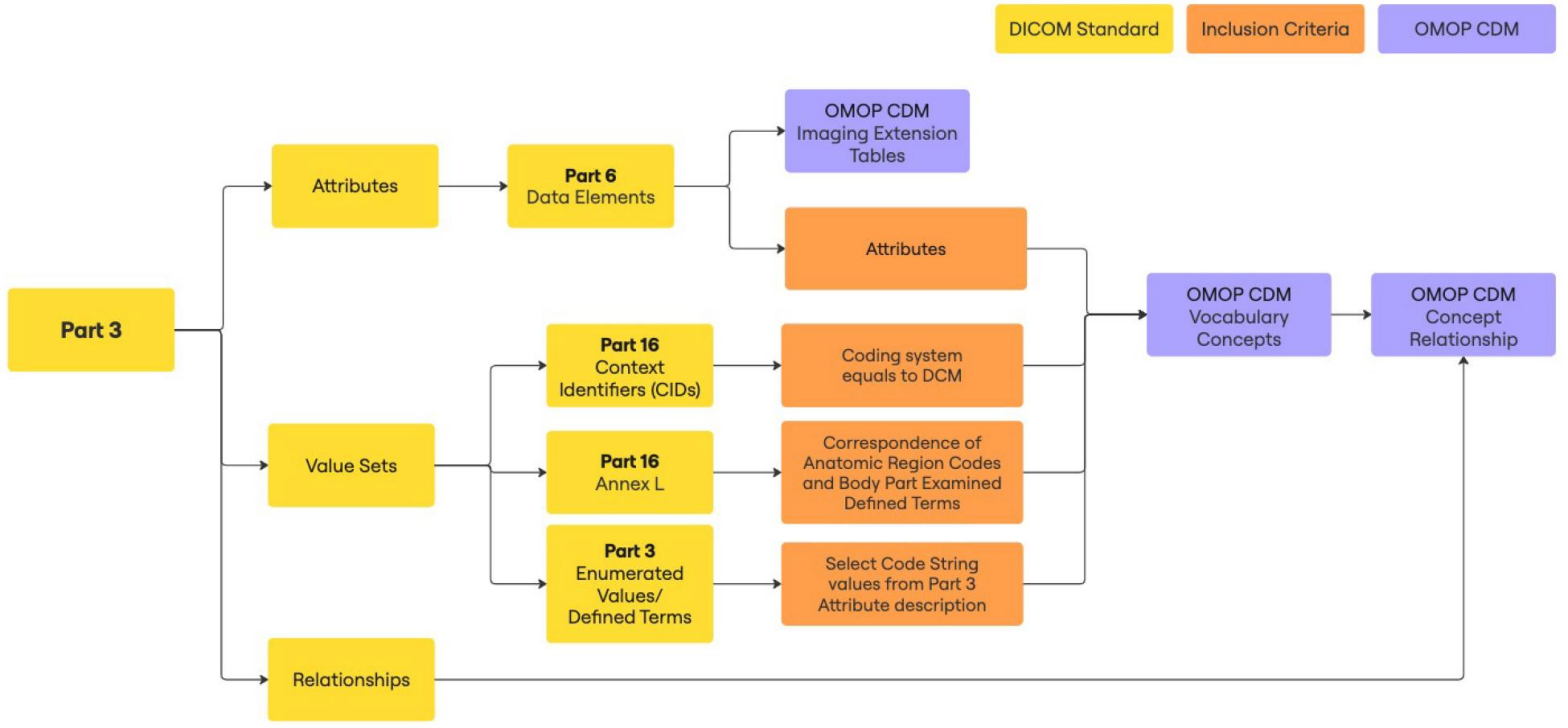
The process diagram from DICOM elements to OMOP CDM vocabulary.

We began by extracting information from DICOM Parts 3, 6, and 16 to define the metadata structure and terminologies necessary for integration into the OMOP CDM. Figure 3 outlines a workflow for extracting, transforming, and loading DICOM terminology into the OMOP CDM Concept, Concept_relationship, and imaging extension tables. Critical identifying Attributes from Part 6 were selected to populate the imaging extension table columns (Table 1). All Attributes (Tags) from Part 6 and the DICOM-managed coded values from Part 16 were added to the Concept table. For other attributes, allowable values are encoded as character strings in Part 3, called “Enumerated Values” or “Defined Terms,” which specify the semantics in the context of that attribute (Appendix S1). We extracted a subset of Enumerated Values and Defined Terms for specific attributes such as *Modality*, *Body Part Examined*, *Patient Position*, and *Lossy Image Compression Method*, and included these in the Concept table. 

**Table 1.** The DICOM attributes for OMOP CDM imaging extension.^a^

MI-CDM tables	MI-CDM columns	DICOM tags	DICOM attribute names	VR	Included as OMOP CDM vocabulary
Image occurrence	image occurrence date	(0008,0021)	*Series Date*	DA	Yes
	image study UID	(0020,000D)	*Study Instance UID*	UI	No
	image series UID	(0020,000E)	*Series Instance UID*	UI	No
	modality concept id	(0008,0060)	*Modality*	CS	Yes
	anatomic site concept id	(0018,0015)	*Body Part Examined*	CS	Yes
Image feature	image feature concept id	All other metadata tags in each row			
	anatomic site concept id	(0018,0015)	*Body Part Examined*	CS	Yes

a The abbreviations used in the table include Value Representation (VR), Date Time (DA), Unique Identifier (as UI in VR and as UID in the column name), and Code String (CS).

These DICOM concepts were subsequently used to populate two types of relationships in the Concept_relationship table: “Maps to” and “Maps to value”.^8^ This convention is used in OMOP CDM to represent the transition from “non-standard” to “standard” concepts. The “Maps to value” relationship captured the mappings between Attributes and their coded values, while the “Maps to” relationship represented mappings between DICOM codes and other “standard” coding systems, such as SNOMED. For example, *Body Part Examined* Defined Terms are mapped to SNOMED codes in Part 16.

To support the integration, we assigned custom concept identifiers (IDs) for the DICOM terminology. The OMOP CDM adopts standardized coding systems, eg, LOINC or SNOMED, mapped to internal concept IDs. Thus, for many of the SNOMED and LOINC codes that appear in DICOM CIDs in Part 16, no custom concept IDs were added. The custom concepts are considered “non-standard” in OMOP CDM and designed to fit specific research needs. In alignment with the OMOP convention, all custom concept IDs for DICOM terminology were assigned to the range starting with 2 128 000 000 as OMOP CDM reserves custom IDs to fall within the 2-billion range.

### Demonstrating the integrated vocabulary

We used the integrated DICOM concepts to demonstrate their practical application in Alzheimer's disease research. ADNI is a data set aimed at identifying the progression of Alzheimer's Disease (AD).^38^ ADNI was originally developed to test whether serial magnetic resonance imaging (MRI), positron emission tomography (PET), other biological markers, and clinical and neuropsychological assessment can be combined to measure the progression of mild cognitive impairment (MCI) and early Alzheimer's disease (AD). This research has expanded to include validating biomarkers for clinical trials, improving the generalizability of ADNI data by increasing diversity in the participant cohort, and providing data concerning the diagnosis and progression of Alzheimer's disease to the scientific community. In this work, we used imaging data from ADNI 3, which includes data collected between 2016 and 2022. This phase emphasizes the use of tau PET and functional imaging in clinical trial settings and includes additional cohorts comprising 133 cognitively normal elderly controls, 151 individuals with MCI, and 87 patients with AD.^39^

To simulate EHR data, we downloaded CSV files for the ADNI data dictionary, patient demographics, diagnoses, and neuropsychological inventory scores. We transformed the downloaded data values and loaded them into the Person, Condition_occurrence, and Measurement tables. Then, we ingested MRI studies from ADNI 3 and extracted metadata from one image in each series. We first extracted the Attributes required to populate the Image_occurrence and Procedure_occurrence tables from imaging studies. After excluding Attributes containing empty values, we selected from the remaining Attributes based on Value Representation (VR) to include any with numeric and coded values, specifically Attribute Tag (AT), Code String (CS), Date (DA), Date Time (DT), Decimal String (DS), Floating Point Single (FL), Floating Point Double (FD), Integer String (IS), Signed Long (SL), Signed Short (SS), Signed 64-bit Very Long (SV), Time (TM), Unsigned Long (UL), Unsigned Short (US), and Unsigned 64-bit Very Long (UV). We avoided free text fields with one exception, *Manufacturer (short text)*, which was needed for cohort definition (Appendix S3). Each filtered Attribute was transformed and loaded to the Image_feature and Measurement tables.

We used OHDSI ATLAS, the web-based cohort discovery tool powered by the populated database to define a cohort of patients with T1-weighted volumetric MRI scans. The inclusion criteria included the key metadata Attributes based on the protocols described by the ADNI project—repetition time (TR), echo time (TE), and inversion time (TI). The computed cohort definition was downloaded as an SQL file to extract a list of DICOM Series UIDs meeting the criteria.

The extraction and transformation codes were written in Python and can be found on the GitHub Page: https://github.com/paulnagy/DICOM2OMOP/.

## Results

### Extracting the DICOM standardized elements and adding DICOM concepts

We extracted 5190 Attributes from Part 6 from the DICOM Standard and, after removing seven Attributes without Attribute Names, assigned 5183 Attributes OMOP CDM concept IDs. We harvested 5223 DICOM-managed coded values from Context Groups and 398 elements from *Body Part Examined* in Part 16. Context Group organizes coded values by Context Group Identifier (CID) with some codes belonging to multiple CIDs. We found 1063 coded values were repeated and selected 3281 unique codes. The other coded values extracted from Part 3 and Part 16 resulted in 79 code string values in *Modality*, 318 in *Body Part Examined*, 16 in *Patient Position*, and 8 in *Lossy Image Compression Method*. Among the 79 *Modality* elements, 74 of them were also found in the Context Group values and removed to prevent duplication. Combining coded values from Parts 3 and 16, we converted 3628 coded values (Table 2).

**Table 2.** Summary of OMOP concepts for DICOM terminology and relationship.

Data type	Data source	Data subtype	Inclusion criteria	No. of OMOP concepts
Attributes	Parts 6	DICOM Data Elements	All	5183
	Total Attributes			**5183**
Coded values	Part 16	Context Identifiers (CID)	Coding system equals to DCM	3281
		*Body Part Examined*	DICOM code string not missing	318
	Part 3	Defined Terms	*Modality*	5
			*Patient Position*	16
			*Lossy Image Compression Method*	8
	Total Coded Values			**3628**
Relationships	Part 3	Attribute to Coded Values		7840
		DICOM Value to Standard Coding System		307
	Total Relationship concepts			**8147**
Total OMOP Concepts for DICOM				**16 958**

Sub-total and grand total values are bolded for readability.

From Part 3, we extracted Attributes and value constraints (expressed through CID or as Enumerated Values or Defined Terms). Using the custom OMOP concepts assigned and Part 16, we identified 7101 “Maps to value” relationships between Attribute and mapped coded values and 739 from Defined Terms*. Body Part Examined* Defined Terms had 307 values mapped to SNOMED (Appendix S2).

### Demonstrating the integrated vocabulary

**Figure 4.**
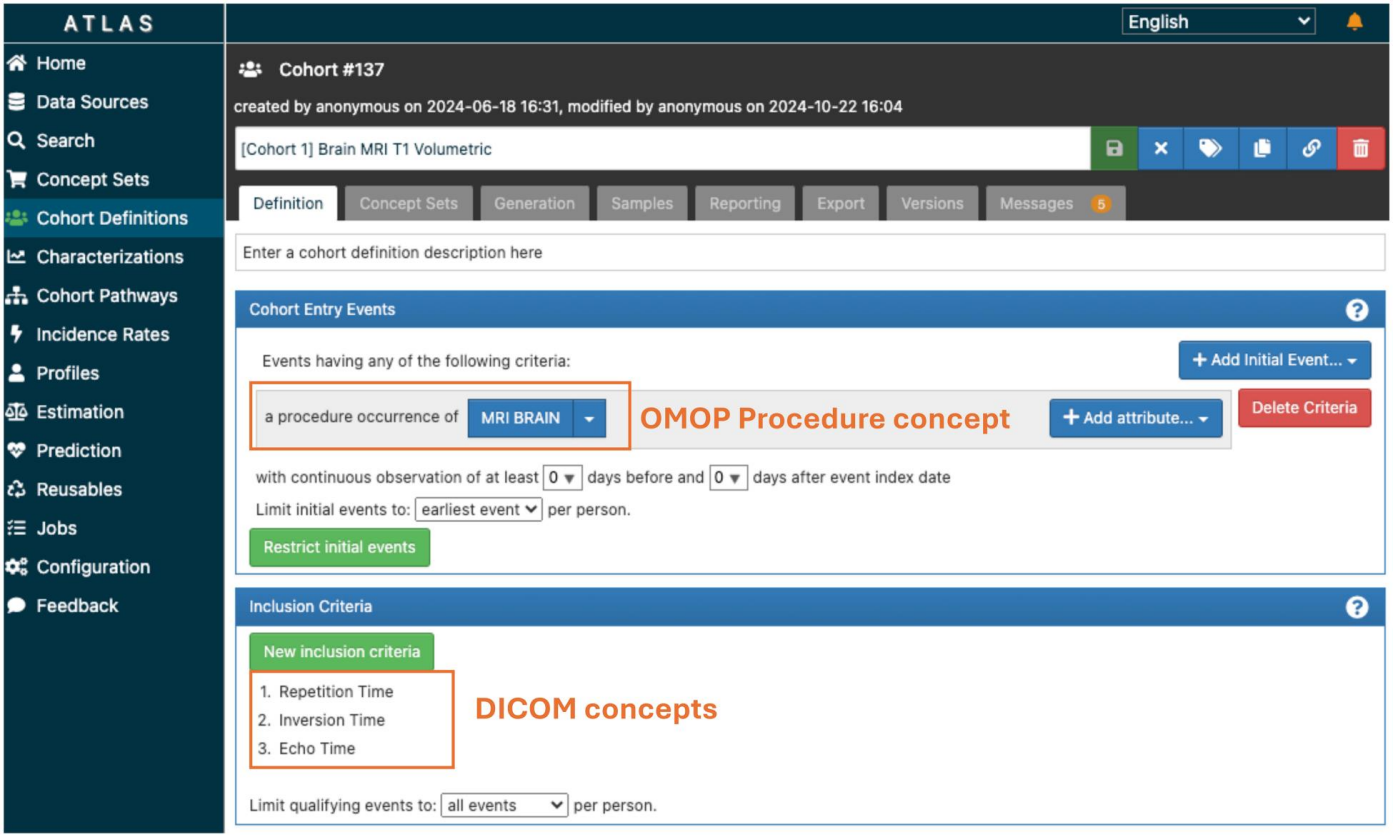
The screenshot of ATLAS demonstration of DICOM concepts.

From the EHR-based ADNI data, we first ingested and uploaded 4152 patients, 8641 diagnoses, and 88 819 answers for Neuropsychiatric inventory responses, which were organized in Person, Condition_occurrence, and Measurement tables. We ingested 545 DICOM studies containing 4756 series from the ADNI database and extracted the DICOM header from one image in each series. We populated the Procedure_occurrence table using study-level metadata and Image_occurrence using series-level metadata. After filtering out the Attributes, we removed attributes containing empty values and added 283 948 metadata elements to the Image_feature and Measurement tables. We created a cohort definition of patients with T1 volumetric brain MRIs, identifying 252 patients (limited by the subset of imaging data). The cohort definition was used to conduct explanatory analysis using the OHDSI ATLAS Characterization tab (Figure 4). The expanded inclusion criteria are provided in Appendix S3.

## Discussion

This work proposes a workflow to integrate DICOM terminology into the OMOP CDM vocabulary, a critical step toward achieving semantic harmonization between medical imaging and clinical data. Incorporating standardized imaging metadata within the OMOP CDM provides the structural framework necessary to computationally and reproducibly characterize imaging studies. With the adoption of imaging extension tables, the OMOP CDM is an important tool for cross-institutional observational studies, enabling reproducible research that can leverage imaging acquisition parameters and analysis features alongside clinical outcomes.

A primary contribution of this work is transforming parts of the DICOM Standard into a compatible vocabulary that can be used in clinical observational research. This model supports detailed and precise phenotype definitions by defining imaging characteristics—such as refining “Brain MRI” to a more specific definition with imaging parameters like “T1-weighted 3D MRI series”. This standardized approach makes phenotypes more easily searchable and reproducible across diverse data sources. Existing data silos hinder researchers from effectively utilizing both EHR data and medical images. In most cases, when conducting an imaging study linked to clinical outcomes, researchers must reach out to the imaging and clinical data managers individually. However, when both data streams are organized in a standardized framework, they can use tools like ATLAS to explore the cohort definitions, observe aggregate counts, and tailor the phenotype before requesting data extraction for EHR data and images.

One of the key strengths of this approach is its easy adoption. The OMOP CDM is already extensively utilized across healthcare institutions globally, establishing it as a reliable platform for incorporating imaging studies. By using an OMOP CDM extension, researchers can access established tools like ATLAS for cohort definition and visualization and HADES for large-scale analytics. This simplifies the integration of imaging data into research. Additionally, computational phenotypes can be exported and reused at any center with the OMOP CDM. Moreover, the OHDSI Evidence Network provides a federated framework for data standardization and analytics, offering researchers a well-established foundation to conduct large-scale imaging and multimodal studies.

One of the limitations of the current model is its reliance on the quality and completeness of input data. For DICOM metadata to be useful, the values within the DICOM tags must be accurate and consistently populated. There is significant variability in how different manufacturers populate DICOM fields, which can lead to discrepancies in data quality.^40^^,^^41^ For example, anatomical description may be contained in up to three different Attributes: *Body Part Examined*, *Anatomic Region Sequence*, and/or *Primary Anatomic Structure*. In this study, we extracted the mappings between Attributes, allowed coded values from Part 3, and loaded them as “Maps to value” in Concept_relationship table. This can be used to evaluate aspects of real-world data's conformance to the DICOM Standard and ensure data quality within the standardized database.

Another potential challenge is the scalability of this model in real-world healthcare environments. This small example captured an average of 61 DICOM metadata elements per imaging series across 289 patients. While this scale was manageable, the larger volume of images and data typical in an EHR database could substantially increase the OMOP database size. Future studies should focus on identifying the most research-relevant DICOM Attributes, which can be prioritized for storage and analysis, thereby balancing the need for comprehensive metadata with concerns about database scalability.

There are also several elements not yet included in the current iteration of the model. For example, the text values from Attributes, such as Long String fields, which are important for capturing information like *Series Descriptions*, have not yet been converted into a standard coding system. Additionally, the integration of Defined Terms and Enumerated Values from DICOM metadata is still incomplete, and private DICOM tags, which contain manufacturer-specific data, were not incorporated. However, the private tags can be standardized following the same workflow as public tags to accommodate specific research goals. Moreover, while this study focused on integrating imaging metadata through DICOM terminology, the quantification of image findings (eg, volumetric measurements or radiomic features) and clinical findings (eg, nodules, solid) through other standard terminology like RadLex and Radiology Common Data Elements have not yet been explored. Future work can expand the current study to investigate integrating other imaging-specific standard vocabularies, providing a more comprehensive picture of imaging's role in clinical research.

## Conclusion

In this work, we demonstrated the feasibility and importance of integrating DICOM terminology into the OMOP CDM vocabulary, leading the way for semantic integration between medical imaging and clinical data. By standardizing imaging metadata and leveraging the OMOP CDM infrastructure, this approach supports reproducible, scalable, and multimodal research across institutions. These advancements bridge a critical gap in linking imaging and clinical datasets, enabling more precise phenotype definitions and fostering collaboration through shared tools and frameworks. By facilitating multimodal observational research that incorporates both imaging features and clinical outcomes, this study establishes a foundation for innovative approaches to understanding complex diseases and advancing personalized medicine.

## Supplementary Material

ocaf091_Supplementary_Data

## Data Availability

The data underlying this article are available in Alzheimer's Disease Neuroimaging Initiative (ADNI) Image and Data Archive (IDA) at https://ida.loni.usc.edu/login.jsp?project=ADNI. The dataset used in the demonstration of the workflow proposed in the manuscript was derived from sources in the public domain from the ANDI project. Additional information about this dataset is listed in the Acknowledgements section per data provider’s guideline.
