# Supplementary material for: Breaking data silos: incorporating the DICOM imaging standard into the OMOP CDM to enable multimodal research
Source: J Am Med Inform Assoc. 2025 Jul 18;32(10):1533–41. doi: 10.1093/jamia/ocaf091 (PMC12451937; doi:10.1093/jamia/ocaf091)

**APPENDIX**

**Appendix A. DICOM Standard Examples**

DICOM Part 3 (Information Object Definitions) defines the relationships between these keys and values for each Information Object Definition (IOD), an object-oriented data model describing real-world imaging objects such as computed radiography images, ultrasound images, and magnetic resonance images[30] . Each IOD is defined by an IOD Module Table listing required and optional modules, indicated in Usage column (M as Mandatory, and U as Optional). Each Module is defined by a Module Attributes Table listing required and optional attributes and value constraints.

The first table shows the Part 3 IOD table for the MR Image IOD. The second table displays part of the General Study Module table (C.7-3) from Section C.7.2.1 (noted with **♦**). The Attribute Requesting Service Code Sequence references CID 7030 "Institutional Department/Unit/Service" (noted with **Δ**). The third table is sample of the C7030 Context Group in Part 16 that includes 86 codes for departments, such as Pediatric Radiology and Neurology.

DICOM has a formal change management process. As a matter of policy, existing attributes such as those indexed in OMOP have almost never been changed over the last 32 years. New attributes are added every year, often as part of supporting new technologies like functional MRI, photon counting CT, optical coherence tomography, or breast tomosynthesis, but those are typically detailed technical parameters. OMOP will need to monitor those additions and periodically incorporate them. The most updated DICOM standard is available at <http://www.dicomstandard.org>.

| Table A.4-1. MR Image IOD Modules | | | |
| --- | --- | --- | --- |
| IE | Module | Reference | Usage |
| Patient | Patient | C.7.1.1 | M |
|  | Clinical Trial Subject | C.7.1.3 | U |
| Study | General Study | **C.7.2.1 ♦** | M |
|  | Patient Study | C.7.2.2 | U |
|  | Clinical Trial Study | C.7.2.3 | U |
| Series | General Series | C.7.1.1 | M |
|  | Clinical Trial Series | C.7.2.3 | U |
| Frame of Reference | Frame of Reference | C.7.4.1 | M |
| Equipment | General Equipment | C.7.5.1 | M |
| Acquisition | General Acquisition | C.7.10.1 | N |
| Image | General Image | C.7.6.1 | M |
|  | General Reference | C.12.4 | U |
|  | Image Plane | C.7.6.2 | M |
|  | Image Pixel | C.7.6.3 | M |
|  | Contrast/Bolus | C.7.6.4 | C – Required if contrast media was used in this image |
|  | Device | C.7.6.12 | U |
|  | Specimen | C.7.6.22 | U |
|  | MR Image | C.8.3.1 | M |
|  | Overlay Plane | C.9.2 | U |
|  | VOI LUT | C.11.2 | U |
|  | SOP Common | C.12.1 | M |
|  | Common Instance Reference | C.12.2 | U |

| **♦Table C.7-3. General Study Module Attributes** | | | |
| --- | --- | --- | --- |
| Attribute Name | Tag | Type | Attribute Description |
| Study Instance UID | (0020,000D) | 1 | Unique identifier for the Study |
| Study Date | (0008,0020) | 2 | Date the Study started |
| Study Time | (0008,0030) | 2 | Time the Study started |
| Referring Physician's Name | (0008,0090) | 2 | Name of the Patient's referring physician |
| Referring Physician Identification Sequence | (0008,0096) | 3 | Identification of the Patient's referring physician. Only a single item is permitted in this Sequence |
| *>Include Table 10-1 "Person Identification Macro Attributes"* | | | |
| Consulting Physician's Name | (0008,009C) | 3 | Consulting physician(s) for this Patient Visit |
| Consulting Physician Identification Sequence | (0008,009D) | 3 | Identification of the consulting physician(s). One or more items are permitted in this Sequence. If more than one item, the number and order shall correspond to the value of Consulting Physician's Name (0008,009C), if present. |
| … | … | … | … |
| Requesting Service | (0032,1033) | 3 | Institutional department, unit or service where the request initiated. See Note 1 and Note 2. |
| Requesting Service Code Sequence | (0032,1034) | 3 | Institutional department, unit or service where the request initiated. Only a single item is permitted in this Sequence |
| *>Include Table 8.8-1 "Code Sequence Macro Attributes"* | | | **Δ *DCID 7030 "Institutional Department/Unit/Service"*** |

| **Δ Table CID 7030. Institutional Department/Unit/Service** | | | | |
| --- | --- | --- | --- | --- |
| Coding Scheme Designator | Code Value | Code Meaning | SNOMED-RT ID | UMLS Concept Unique UD |
| DCM | 128177 | Pediatric Radiology |  |  |
| SCT | 309937004 | Neurology | R-3025E | C0587475 |

**Appendix B. Concept Relationship Table Examples.**

Examples of DICOM concept in OMOP CDM Concept and Concept relationship tables. The DICOM Attributes and Coded Values were added to the Concept table, which is the vocabulary table in OMOP CDM. The Concept Code stores the original Attribute tags or DCM codes. The Concept relationship table includes two types of relationships for DICOM concepts: “Maps to value” to link Attributes and Coded Values, and “Maps to” to map DICOM code to other standard vocabulary, such as SNOMED.

| OMOP CDM Concept Table | | | | |
| --- | --- | --- | --- | --- |
| Concept ID | Concept Name | Vocabulary ID | Concept Class ID | Concept Code |
| 2128000809 | Slice Thickness | DICOM | DICOM Attributes | 00180050 |
| 2128000002 | Specific Character Set | DICOM | DICOM Attributes | 00080005 |
| 2128000003 | Language Code Sequence | DICOM | DICOM Attributes | 00080006 |
| 2128006300 | Curve Fitted Reference | DICOM | DICOM Value Sets | 122489 |
| 2128006303 | Distance | DICOM | DICOM Value Sets | 121206 |
| 2128006310 | Image container label | DICOM | DICOM Value Sets | 121354 |

| OMOP CDM Concept Relationship Table | | | | |
| --- | --- | --- | --- | --- |
| Concept ID 1 | Concept ID 2 | Relationship ID | Concept 1 Name* | Concept 2 Name* |
| 2128000784 | 2128021398 | Maps to value | Body Part Examined | ABDOMEN |
| 2128000323 | 4184966 | Maps to value | Ethnic Group Code Sequence | American Indian or Alaska Native |
| 2128002343 | 4150861 | Maps to value | Requesting Service Code Sequence | Psychology service |
| 2128021398 | 37303869 | Maps to | Abdomen | Cross-sectional abdomen |
| 2128021430 | 4133034 | Maps to | Brain | Brain Structure |

* Concept names are not part of the Concept Relationship table; added for readers’ understanding.

**Appendix C. Screenshots from the ATLAS Cohort Discovery Tool.**

The first picture is from the cohort definition inclusion criteria panel, which shows that DICOM Attributes and values can be used to specify the ranges for the repetition time. The inclusion criteria based on three Attributes: repetition time (TR), echo time (TE), and inversion time (TI), which vary among the three main MRI manufacturers. Siemens typically uses a TR of 2300 ms, TE of 2.98 ms, and TI of 900 ms for high-resolution anatomical imaging. Philips employs a shorter TR of 6.5 ms, TE of 2.9 ms, and TI of 870 ms, optimized for faster acquisition. GE sequences often feature a TR of 2500 ms, TE of less than 10 ms, and TI of 400 ms, balancing rapid tissue contrast and adaptability. These differences reflect each manufacturer's proprietary technologies and necessitated accommodating a range of values in the cohort inclusion criteria.

The second picture is a part of the Characterization tab from the ATLAS, where the users can conduct exploratory analysis on the cohort using covariates such as Neuropsychiatric inventory score by sub-group. The sub-group is also defined within the ATLAS platform using the diagnosis codes—Clinically Normal, Dementia, and Minor Cognitive Impairment.


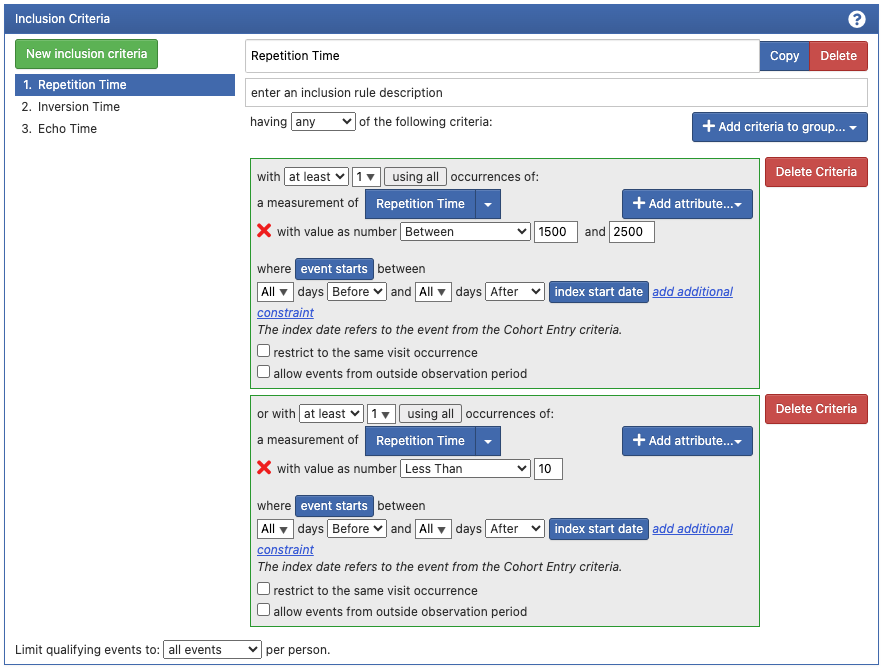


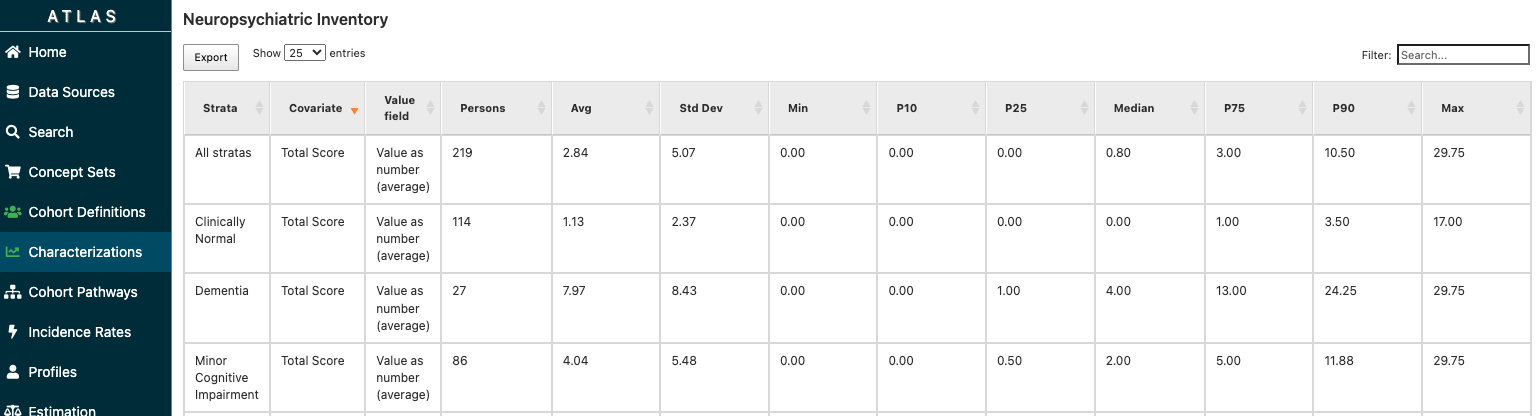

Supplement: ocaf091_Supplementary_Data [file ocaf091_supplementary_data.docx]
